# When cost-effective interventions are unaffordable: Integrating cost-effectiveness and budget impact in priority setting for global health programs

**DOI:** 10.1371/journal.pmed.1002397

**Published:** 2017-10-02

**Authors:** Alyssa Bilinski, Peter Neumann, Joshua Cohen, Teja Thorat, Katherine McDaniel, Joshua A. Salomon

**Affiliations:** 1 Interfaculty Initiative in Health Policy, Harvard University, Cambridge, Massachusetts, United States of America; 2 Center for Evaluation and Risk in Health, Tufts Medical Center, Boston, Massachusetts, United States of America; 3 School of Social and Political Science, University of Edinburgh, Edinburgh, Scotland, United Kingdom; 4 Center for Health Policy and the Center for Primary Care and Outcomes Research, Stanford University, Stanford, California, United States of America; 5 Department of Global Health and Population, Harvard T.H. Chan School of Public Health, Boston, Massachusetts, United States of America

## Abstract

Potential cost-effective barriers in cost-effectiveness studies mean that budgetary impact analyses should also be included in post-2015 Sustainable Development Goal projects says Joshua Salomon and colleagues.

**Summary points**

- Many health interventions deemed cost-effective are not affordable. Despite the importance of affordability to policymakers, little of the cost-effectiveness literature in global health addresses this issue.
- Budget impact analysis (BIA) describes an intervention’s short-term costs and savings from the payer’s perspective.
- Researchers should report BIA alongside cost-effectiveness analysis (CEA). When CEA and BIA lead to different conclusions, researchers should explain why.
- Policymakers should recognize that not all cost-effective interventions are affordable and interpret information about cost-effectiveness in the context of their budget and other available funding sources.
- Both cost-effectiveness and affordability should be reflected in the design of essential health service packages.

## Introduction

The post-2015 Sustainable Development Goals call for governments to combat infectious disease, reduce maternal and infant mortality, and ensure that quality healthcare is accessible and affordable to all [1]. To meet these objectives, about half of all countries are in the midst of efforts to introduce or extend universal health coverage (UHC) [2]. This process requires governments to define essential service packages guaranteed to all citizens. Because of resource limitations, these packages cannot include all health services. As a result, both researchers and policymakers have recommended prioritizing cost-effective interventions [3–5].

However, cost-effective interventions are not always affordable. In some cases, adopting cost-effective interventions would necessitate eliminating other, more beneficial expenditures. In a highly publicized example, new medications for chronic hepatitis C were found to be cost-effective in many settings, even at high prices [6–8], but provision of these medications to all potential beneficiaries has been unaffordable, even with discounts [9,10]. Affordability challenges have also arisen with numerous other interventions, including vaccines for human papillomavirus (HPV) and pneumococcal infections [11,12] and GeneXpert tuberculosis diagnostics [13,14]. This disconnect between cost-effectiveness and affordability can complicate efforts to identify and adopt high-value programs.

This paper first assesses the current use of budget impact analysis (BIA) and cost-effectiveness analysis (CEA) in health economic assessments conducted for low- and middle-income countries (LMICs) (**Table 1**). We then recommend steps researchers and policymakers can take to better incorporate affordability information into health economic evaluations, alongside CEA.

**Table 1:** Comparison of CEA and BIA.

	CEA	BIA
***Objective***	Quantify an intervention’s net health return on investment	Quantify an intervention’s impact on resources consumed
***Outcomes***	Net health benefits, net resource consumption	Net resource consumption
***Perspective***	Societal, healthcare sector, or payer	Payer
***Time horizon***	Long-term (until all costs and benefits are realized)	Budget cycle (typically 1–5 years)
***Unit***	$ICER=\frac{{Costs}_{intervention}-{Costs}_{comparator}}{{Benefits}_{intervention}-{Benefits}_{comparator}}$	Absolute costs and savings ($)
***Interpretation***	A smaller ICER indicates lower costs per unit of health gained, i.e., greater cost-effectiveness	Lower costs indicate greater affordability
***Threshold***	New intervention is “cost-effective” if ICER falls below a WTP threshold	No standard to evaluate the affordability of each intervention individually

Abbreviations: BIA, budget impact analysis; CEA, cost-effectiveness analysis; ICER, incremental cost-effectiveness ratio; WTP, willingness to pay

## Current state of CEA and BIA

While political, social, and cultural factors play an important role in budget allocation, CEA can inform decisions on how to maximize health returns from limited resources. Over the past decade, interest in evaluating affordability has also increased [9,15,16]. BIA assesses affordability by estimating an intervention’s short-term net costs from the payer's perspective [17]. Many countries, including Canada, the United Kingdom, Brazil, and Thailand request both BIA and CEA when assessing whether to include a drug on a public formulary [17–20]. In 2014, the Bill & Melinda Gates Foundation also recommended including both BIA and CEA in health economic assessments [21]. Still, BIA is rarely considered in priority-setting frameworks for UHC [16].

Furthermore, peer-reviewed health economic literature for LMICs often lacks budget impact information. For example, we investigated the use of BIA in articles catalogued in the Tufts Medical Center Global Health Cost-Effectiveness Analysis (GHCEA) Registry. The GHCEA Registry contains information on all peer-reviewed English-language CEA articles with health benefits measured in terms of averted disability-adjusted life years (DALYs) [22]. We found that only 3% (*n*/*N* = 12/384) of the articles in the GHCEA conducted a formal BIA, explicitly mentioning BIA in the methods and results sections. Another 10% of the articles (*n*/*N* = 37/384) informally included some measure of budget impact, often in the discussion section. (See S1 Text for inclusion criteria and S1 Table for articles included.)

**Fig 1:**
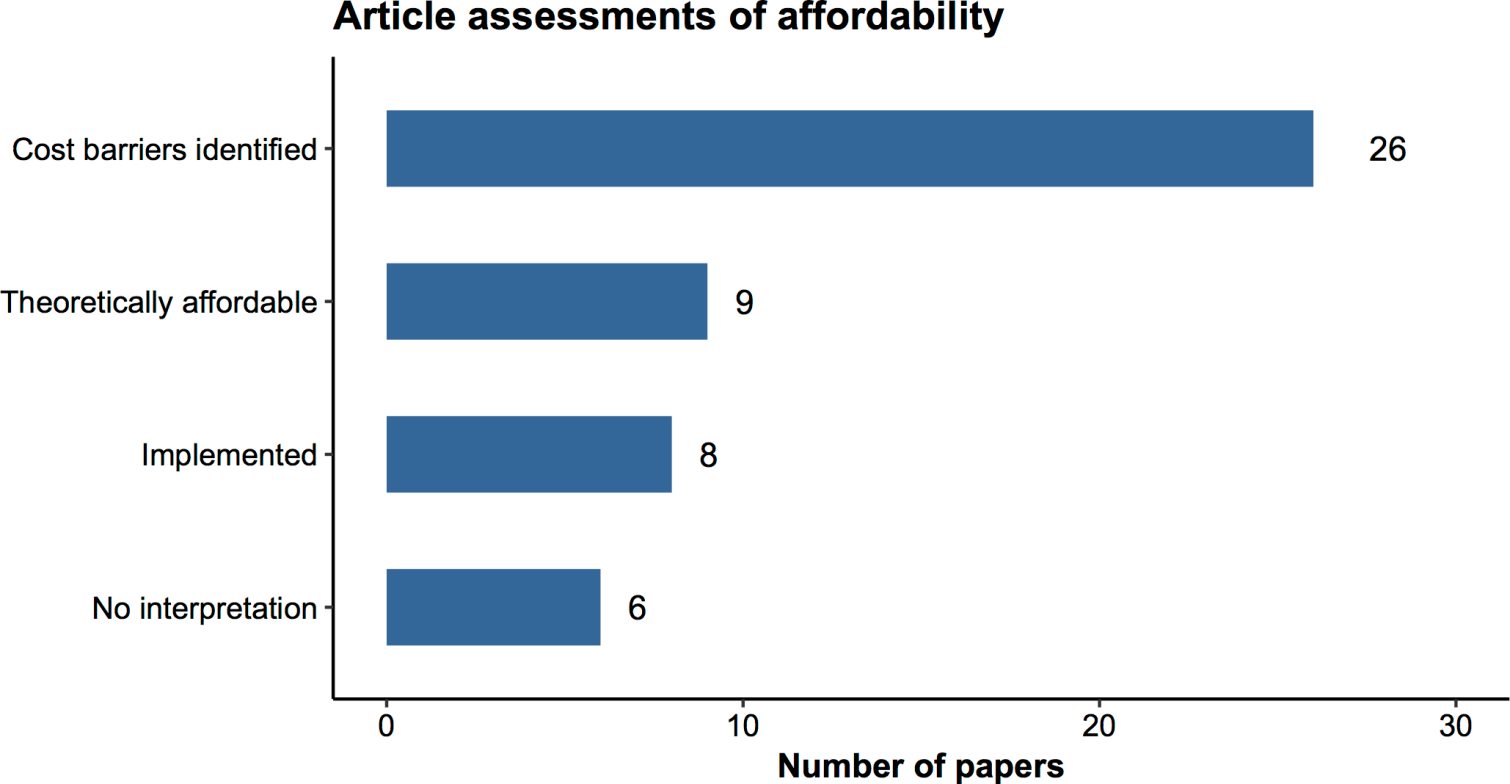
Assessments of affordability in the cost-effectiveness literature. Categories reflect author assessments of affordability based on BIA results. “Cost barriers identified” indicates that the author identified one or more factors that might render an intervention unaffordable. “Theoretically affordable” interventions were deemed feasible with current resources and/or available external support (e.g., Gavi funding). “Implemented” interventions had already been implemented at time of publication. “No interpretation” indicates that the author conducted a BIA but did not make statements about the intervention’s affordability. Examples of how we classified assessments appear in S2 Text. Data Source: author extraction from the GHCEA Registry (www.ghcearegistry.org). Abbreviations: BIA, budget impact analysis; GHCEA, Global Health Cost-Effectiveness Analysis.

When articles presented both CEA and BIA, their recommendations often diverged. More than half of the articles that reported formal or informal BIA findings concluded that cost-effective interventions might be unaffordable (**Fig 1)**. One stated, “The financial realities facing resource-deprived health systems in developing countries make it impossible to carry out all potentially ‘very cost-effective’ interventions” [23]. Others concluded that program budgets [24] or even national health budgets [25] would have to be tripled or quadrupled in order to accommodate cost-effective interventions.

Some articles suggested price reductions to address affordability issues [12,26,27]. One wrote, “hopefully, [the price of the rotavirus vaccine] will be reduced in light of this analysis” [28], and another recommended a stronger drug negotiation policy for cholera vaccines [23]. Others suggested that additional resources from external funders could subsidize program costs [12,26,29]. For example, for a malaria home management program, an article stated that “set up costs may be particularly suitable for funding by donor organizations…while subsequent costs could be contained within the budget of a typical sub-Saharan African District” [30].

## Next steps forward: Reconciling cost-effectiveness and affordability

### How can cost-effective interventions be unaffordable?

The gap between cost-effectiveness and affordability can be confusing because CEA appears to account for affordability: it benchmarks an intervention’s value against a measure of social willingness to pay (WTP) for health improvements (**Table 1**). However, CEA addresses affordability only indirectly and incompletely.

In theory, CEA assumes that a policymaker would conduct a “shopping spree” with a fixed budget and information about the cost-effectiveness of all available programs. In order to decide which programs to fund, the policymaker would first rank programs by incremental cost-effectiveness ratio (ICER) (**Table 1**), a measure of cost-effectiveness. The policymaker would then adopt programs in order of cost-effectiveness, continuing until the health budget is exhausted. Because the “shopping spree” continues only until the budget is exhausted, the set of selected programs is, by definition, affordable. The ICER of the last program adopted (i.e., the least cost-effective program in the budget) is designated the WTP threshold. Programs with ICERs lower than the threshold are both cost-effective and affordable.

In practice, however, CEA articles typically consider only a few interventions and do not conduct a “shopping spree.” Instead, the cost-effectiveness threshold is identified exogenously. Researchers measure the aggregate cost of death and disability in disability-adjusted life years (DALYs) or quality-adjusted life years (QALYs), both of which reflect longevity and quality of life. Gaining a year of life in perfect health, for example, averts 1 DALY, whereas gaining a year of life in some less-favored health state averts a fraction of a DALY, depending on the severity of the morbidity for that health state. The health economics literature often designates interventions that avert a DALY (or gain a QALY) for no more than 1–3 times a country’s per capita gross domestic product (GDP) as “cost-effective.” This is justified by the claim that a country should be willing to pay about as much for a life year as an average person would produce in that year. In many countries, however, healthcare budgets are not large enough to fund all interventions with ICERs below this ceiling [31]. Therefore, some cost-effective interventions are not affordable.

### Explaining the divergence between affordability and cost-effectiveness

#### Budget size and the WTP threshold

One reason BIA and CEA may diverge is if the presumed WTP threshold in CEA is too high. Suppose a country’s budget is insufficient to support all programs with ICERs below the WTP threshold. If the healthcare budget reflects the preferences of the population regarding the appropriate level of spending on health (or otherwise cannot be increased), then the true WTP threshold must be lower than what is assumed. Ideally, the WTP threshold should reflect current spending, and researchers have begun to estimate empirical thresholds based on a country’s current budget [31].

However, revising the WTP threshold presents several challenges. Empirical thresholds are difficult to calculate because the process requires knowing the cost-effectiveness of all funded programs. In addition, it is common for some unfunded programs to be more cost-effective than programs that are funded. Even if the healthcare budget is theoretically large enough to fund all cost-effective interventions, if part of that budget is diverted to fund interventions that are not cost-effective, then some cost-effective interventions may not be affordable. In complex, rapidly changing health systems, CEA cannot provide simple rules. Even high-value “cost-effective” programs may require more resources than are available in a given budget.

Few LMICs currently meet the WHO spending targets for per capita health spending [32], and several studies in our sample emphasized the need for increasing overall health budgets [25,33]. In some cases, cost-effectiveness evidence can increase political will to dedicate resources to high-value health services. However, large budget increases may not be politically or economically feasible given limited resources. Acknowledging this, some papers recommended starting with higher-value, lower-cost interventions. For example, one article suggested expanding non-radiologic, very cost-effective breast cancer screening programs in Mexico and Costa Rica if substantial new resources could not be marshalled for mammography, or to focus mammography on highest-risk groups [24].

#### Analytical perspective

Some authors attribute the divergence of affordability and cost-effectiveness primarily to differences in how these two approaches estimate WTP values [34]. However, this view minimizes other differences between the two types of analysis. For example, CEAs often take a societal perspective, including costs borne by patients [35], or a healthcare perspective, which includes third-party payments. By contrast, BIA only includes payer costs. The choice of perspective can strongly influence ICER estimates and may highlight cases in which a program alleviates or exacerbates a large financial burden on patients or other segments of society. For example, an analysis of rotavirus vaccination showed that incorporating financial and productivity costs borne by patients decreased the ICER by 30%–75% [36]. Another article found that if cost estimates were reduced by the size of the subsidies provided by Gavi, rotavirus vaccination ICERs decreased by 50% [29].

#### Distribution of costs and benefits

Most guidelines for conducting CEA suggest that cost-effectiveness ratios should be benchmarked against a WTP threshold to assess value for money. If a new intervention has an ICER below (i.e., more favorable than) the threshold, it is theoretically possible to adopt this alternative in place of a less efficient existing program. In practice, this replacement may not be feasible because programs with more favorable ICERs may also make much bigger demands on the budget. For example, replacing a program that costs $1 million and averts 50,000 DALYs ($20 per DALY averted) with a more efficient program that costs $10 million and averts 1 million DALYs ($10 per DALY averted) increases healthcare spending by $9 million. Finding the extra $9 million may not be possible. In addition, some programs may not confer full benefits for decades. Cost-effectiveness incorporates cost offsets, but because such offsets may occur in the distant future, accrue to different programs and payers, and may be uncertain, they are less salient to short-term budget considerations.

Both high costs and delayed cost offsets have been salient to the assessment of new hepatitis C virus (HCV) medications, which are cost-effective but far more expensive than previous HCV medications. One analysis estimated that treating all patients with these drugs would incur costs amounting to 10% of all pharmaceutical expenditures in several countries [10,37]. While the drugs are associated with cost offsets, these offsets do not accrue for decades. As a result, countries that have adopted these medications have often negotiated low prices, even if higher prices were cost-effective (e.g., Egypt) or limited access to the drugs (e.g., the United States) [9,10,38,39].

#### Discounting

A final reason for inconsistencies between CEA and BIA stems from differences in how they treat discounting. CEA discounts costs and benefits, typically at a rate of 3% annually [40,41]. In contrast, BIA guidelines do not recommend discounting because the budget must include full costs and because it is often infeasible to invest a health budget and gain a return [17]. In general, use of discounting makes programs with benefits that occur in the near term look more favorable and programs with delayed benefits (e.g., many preventive programs) look less favorable.

#### Recommendations

**Fig 2:**
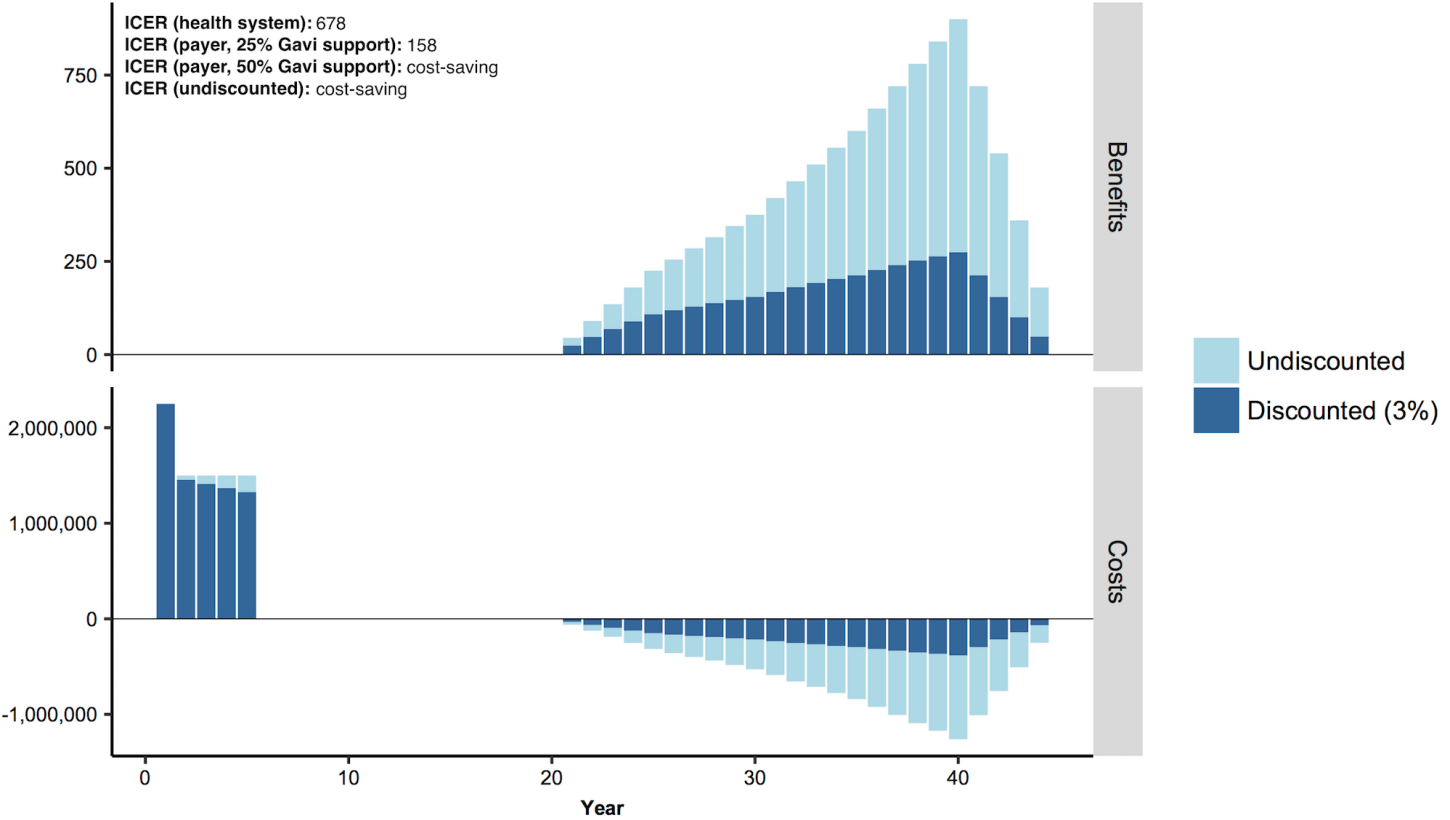
Distribution of costs and benefits per year for a stylized vaccination intervention. Total bar height is the undiscounted cost or benefit. The dark blue portion of the bar is the present value cost or benefit with a 3% annual discount rate. Abbreviation: ICER, incremental cost-effectiveness ratio.

To address the fact that interventions deemed cost-effective in the published literature are not always affordable, we propose including information about implementation costs alongside cost-effectiveness. First, researchers should present BIA alongside CEA. For most health services, there is no budget impact information available. If the size of a program’s eligible population can be estimated, standardized BIA can be readily conducted using information already developed for CEA (see **Fig 2**). At a minimum, this should include cost and cost offsets over a short time horizon; if possible, researchers should benchmark costs against the available local budget.

Second, researchers should highlight possible reasons for divergence between CEA and BIA, which can help to identify and prioritize high-value programs. These can help policymakers interpret evaluations of cost-effectiveness in light of available resources by (1) identifying high-return packages of services conditional on existing budgets and (2) suggesting areas that would yield high benefit from increased investment. In **Table 2**, we summarize these recommendations for research and policy.

**Table 2:** Research and policy/advocacy recommendations for CEA and BIA.

		Research	Policy/Advocacy
**Conducting BIA**		**Add BIA to CEA**	**Request CEA and BIA**
	*Costs and savings*	Report undiscounted payer costs and savings over 1–5 year time horizon in current country currency	***In most cases*, *not all “cost-effective” interventions will fit into the budget*.** Compare the relative cost-effectiveness of different strategies. All else equal, choose interventions with lower ICERs. Aim to reduce spending on interventions with high ICERs, and increase spending on those with low ICERs.
	*Benchmark*	Benchmark cost as a percentage of the current budget	
	*Context*	Indicate programs that might be reduced or eliminated to add new interventions	
**Combining BIA and CEA**		**Compare CEA and BIA**	**Use BIA to inform CEA**
	*Time horizon*	Report costs and benefits accrued per year	Seek external support for programs with favorable ICERs but high upfront costs.
	*Perspective*	Report health sector, societal, and payer ICERs	Identify opportunities for allocating costs across sectors, particularly when benefits are shared among different sectors.
	*Discounting*	Report discounted and undiscounted ICERs	Work with researchers to ensure that discounting reflects local preferences and investment opportunities.

Abbreviations: BIA, budget impact analysis; CEA, cost-effectiveness analysis; ICER, incremental cost-effectiveness ratio.

#### Example

We explore a brief example of how these recommendations might inform research and policy (see S3 Text for derivation). In **Fig 2**, we display costs and benefits per year for a stylized example based on HPV vaccination of 5 cohorts, each of 100,000 10-year-old girls in a low-income country [12,42–44]. While the health system ICER would be cost-effective based on per capita GDP in many countries, all costs are borne upfront while benefits would not be experienced for decades. We estimate a cost of approximately $8.25 million for a 5-year vaccination program, with highest costs in the first year of the program. Few LMICs could support that expense. For example, in 2015, government expenditures on routine immunizations averaged around $9 million across countries in sub-Saharan Africa [45]. Based on this average, and on the yearly costs for a new vaccine in our example, the additional intervention would require a 17%–25% annual budget increase.

To address large upfront investments often required for vaccination, Gavi, the Vaccine Alliance, provides cofinancing for LMICs. These yield country contributions as low as $0.20 per vaccine, with gradual increases until the country independently finances the vaccine. When we recalculated the ICER assuming Gavi covered 25% of the cost, payer ICER decreased from $678/DALY to $158/DALY. If Gavi covered half the cost, the payer ICER would become negative, indicating the intervention is cost-saving, but upfront costs still might not be affordable for some countries.

## Conclusion

Designing high-quality healthcare in the era of universal coverage requires cost-effectiveness and budget impact information for health services in different settings. We found that fewer than 5% of global health CEAs conduct BIAs. With information about both cost-effectiveness and budget impact, policymakers can better develop a high-value set of programs for specific contexts. They can also identify services with high costs but high potential population health benefits for which to seek collaboration or external financial support, particularly preventative services and those that provide long-term cost savings. To promote effective incorporation of economic evidence in decision-making, researchers must address gaps in data and clearly communicate findings to policymakers.

Beyond economic value, there are many additional considerations in budget decisions, including the need to identify complete and accurate costs [46], balance competing priorities [47], incorporate equity and financial protection considerations [48], and operate in health systems with multiple payers [16]. Nevertheless, alongside these factors, rigorous consideration of both cost-effectiveness and affordability should be key elements in the design of packages of essential global health services.

## Supporting information

S1 Table Articles with formal and informal BIA. (PDF)

S1 Text Selection process and summary of articles analyzed. (PDF)

S2 Text Examples of classifications in Fig 1. (PDF)

S3 Text Derivation of stylized HPV vaccine example. (PDF)

## Abbreviations

BIA: budget impact analysis
CEA: cost-effectiveness analysis
DALY: disability-adjusted life year
GDP: gross domestic product
GHCEA: Global Health Cost-Effectiveness Analysis
HCV: hepatitis C virus
HPV: human papillomavirus
ICER: incremental cost-effectiveness ratio
LMICs: low- and middle-income countries
QALY: quality-adjusted life year
UHC: universal health coverage
WTP: willingness to pay
