# Supplementary material for: When cost-effective interventions are unaffordable: Integrating cost-effectiveness and budget impact in priority setting for global health programs
Source: PLoS Med. 2017 Oct 2;14(10):e1002397. doi: 10.1371/journal.pmed.1002397 (PMC5624570; doi:10.1371/journal.pmed.1002397)
Supplement: S1 Table — (PDF) [file pmed.1002397.s001.pdf]

S1 Table: Articles with formal and informal BIA

A Bilinski, P Neumann, J Cohen, T Thorat, K McDaniel, JA Salomon

GHCEA Registry Article ID	PubMed ID	Title	Year	Categorization
2002-01-0515	12076553	Feasibility and cost-effectiveness of standardised second-line drug treatment for chronic tuberculosis patients: a national cohort study in Peru.	2002	Informal
2003-01-0408	14997239	Human health benefits from livestock vaccination for brucellosis: case study.	2003	Informal
2005-01-00265	16282407	Cost effectiveness analysis of strategies for maternal and neonatal health in developing countries.	2005	Informal
2005-01-00333	15689430	The cost-effectiveness of introducing hepatitis B vaccine into infant immunization services in Mozambique.	2005	Informal
2006-01-00200	16968123	Feasibility and cost-effectiveness of treating multidrug-resistant tuberculosis: a cohort study in the Philippines.	2006	Informal
2006-01-00214	16785697	Cost-effectiveness analysis of antiretroviral drug treatment and HIV-1 vaccination in Thailand.	2006	Informal
2006-01-00222	16682433	The cost-effectiveness of improving malaria home management: shopkeeper training in rural Kenya.	2006	Informal

2006-01-00251	16317205	Cost-effectiveness of free HIV voluntary counseling and testing through a community-based AIDS service organization in Northern Tanzania.	2006	Informal
2007-01-00072	18038073	Economic evaluation of hepatitis B vaccination in low-income countries: using cost-effectiveness affordability curves.	2007	Informal
2007-01-00105	17875014	Cost-effectiveness of annual targeted larviciding campaigns in Cambodia against the dengue vector Aedes aegypti.	2007	Informal
2007-01-00135	17535105	Cost-effectiveness of rapid syphilis screening in prenatal HIV testing programs in Haiti.	2007	Informal
2007-01-00167	17308266	What is the most cost-effective population-based cancer screening program for Chinese women?	2007	Informal
2008-01-00031	18398383	Vaccine-preventable haemophilus influenza type B disease burden and cost-effectiveness of infant vaccination in Indonesia.	2008	Informal
2008-01-01783	18562457	The cost of Child Health Days: a case study of Ethiopia's Enhanced Outreach Strategy (EOS).	2008	Informal
2009-01-01591	19931723	Economic evaluation of a routine rotavirus vaccination programme in Indonesia.	2009	Informal
2009-01-01612	19824189	Cost-effectiveness of new-generation oral cholera vaccines: a multisite analysis.	2009	Informal
2009-01-01613	19817621	Cost-benefit analysis of a rotavirus immunization program in the Arab Republic of Egypt.	2009	Informal
2009-01-01617	19817591	Cost-effectiveness of rotavirus vaccination in peru.	2009	Informal
2009-01-01633	19706492	Transmission dynamics and economics of rabies control in dogs and humans in an African city.	2009	Informal
2009-01-01712	19159483	Cost-effectiveness of Rotavirus vaccination in Vietnam.	2009	Informal
2010-01-01406	21179503	The potential economic value of a Trypanosoma cruzi (Chagas disease) vaccine in Latin America.	2010	Formal

2010-01-01517	20470426	Health and economic impact of rotavirus vaccination in GAVI-eligible countries.	2010	Formal
2011-01-01260	21945959	The cost-effectiveness of rotavirus vaccination in Armenia.	2011	Formal
2011-01-01287	21857810	Serological testing versus other strategies for diagnosis of active tuberculosis in India: a cost-effectiveness analysis.	2011	Informal
2011-01-01307	21734764	Cost-effectiveness of parenteral artesunate for treating children with severe malaria in sub-Saharan Africa.	2011	Formal
2011-01-01312	21720546	Comparative economic evaluation of Haemophilus influenzae type b vaccination in Belarus and Uzbekistan.	2011	Informal
2012-01-01089	22719233	A multifaceted intervention to improve the quality of care of children in district hospitals in Kenya: a cost-effectiveness analysis.	2012	Formal
2012-01-01374	21378101	Cost-effectiveness of community-based management of acute malnutrition in Malawi.	2012	Informal
2013-01-00734	24349314	Cost-effectiveness analysis of breast cancer control interventions in Peru.	2013	Formal
2013-01-00744	24331749	Model-based impact and cost-effectiveness of cervical cancer prevention in sub-Saharan Africa.	2013	Formal
2013-01-00773	24223524	Antenatal Syphilis screening using point-of-care testing in Sub-Saharan African countries: a cost-effectiveness analysis.	2013	Formal
2013-01-01153	22407018	Cost-effectiveness of Haemophilus influenzae type b (Hib) vaccine introduction in the universal immunization schedule in Haryana State, India.	2013	Informal
2014-01-00629	24769920	Cost-effectiveness of breast cancer control strategies in central america: the cases of costa rica and Mexico.	2014	Formal
2014-01-00988	23242696	Child Health Week in Zambia: costs, efficiency, coverage and a reassessment of need.	2014	Informal
2014-01-02096	25476586	Cost-effectiveness of larviciding for urban malaria control in Tanzania.	2014	Informal

2014-01-02136	25304420	Cost-effectiveness of HIV prevention for high-risk groups at scale: an economicevaluation of the Avahan programme in south India.	2014	Informal
2014-01-02222	24969782	Scaling up integrated prevention campaigns for global health: costs andcost-effectiveness in 70 countries.	2014	Informal
2015-01-01801	26491060	Scaling-up essential neuropsychiatric services in Ethiopia: a cost-effectiveness analysis.	2015	Formal
2015-01-01824	26423002	Scaling up of HIV treatment for men who have sex with men in Bangkok: a modellingand costing study.	2015	Informal
2015-01-01827	26413788	Improving Maternal Care through a State-Wide Health Insurance Program: A Cost andCost-Effectiveness Study in Rural Nigeria.	2015	Formal
2015-01-01957	25919162	Cost-effectiveness of rotavirus vaccination in Albania.	2015	Informal
2015-01-01960	25919158	Cost-effectiveness of HPV vaccination in Belize.	2015	Informal
2015-01-01961	25919157	Cost-effectiveness analysis of the introduction of the human papillomavirusvaccine in Honduras.	2015	Formal
2015-01-01962	25919156	Cost-effectiveness analysis of 10- and 13-valent pneumococcal conjugate vaccines in Peru.	2015	Informal
2015-01-01963	25919154	Cost-effectiveness analysis of introducing universal human papillomavirusvaccination of girls aged 11 years into the National Immunization Program inBrazil.	2015	Informal
2015-01-02007	25768008	A cost-effectiveness analysis of a program to control rheumatic fever andrheumatic heart disease in Pinar del Rio, Cuba.	2015	Informal
2015-01-02030	25691915	Comparison of impact and cost-effectiveness of rotavirus supplementary androutine immunization in a complex humanitarian emergency, Somali case study.	2015	Informal

2015-01-02335	26555122	Health and Economic Implications of National Treatment Coverage forCardiovascular Disease in India: Cost-Effectiveness Analysis.	2015	Informal
2016-01-02270	26765291	Health and economic benefits of public financing of epilepsy treatment in India: An agent-based simulation model.	2016	Informal
