# Supplementary material for: When cost-effective interventions are unaffordable: Integrating cost-effectiveness and budget impact in priority setting for global health programs
Source: PLoS Med. 2017 Oct 2;14(10):e1002397. doi: 10.1371/journal.pmed.1002397 (PMC5624570; doi:10.1371/journal.pmed.1002397)
Supplement: S1 Text — (PDF) [file pmed.1002397.s002.pdf]

S1 Text: Selection process and summary of articles analyzed

A Bilinski, P Neumann, J Cohen, T Thorat, K McDaniel, JA Salomon

Selection process

We first used a full-text search on the Global Health Cost-Effectiveness Analysis (GHCEA) Registry (n=484) to identify articles from low-and-middle income countries (LMIC) (n=384). We then found articles either contained the phrase "budget impact" in a full-text search (n=19) and/or were marked in the GHCEA Registry as presenting aggregate cost for a full target population (n=34). From this subset, we identified studies that conducted a formal BIA (n=12), which mentioned BIA in their methods and results sections or informal BIA (n=37), mentioning budget impact in the discussion section or indirectly in other results.

Summary of articles with formal or informal BIA

**Category**	**Item**	**Formal BIA articles (N = 12)**	**All LMIC articles (N = 384)**
Intervention[1]	Immunization (%)	25 (51)	118 (31)
	Pharmaceutical or device (%)	14 (29)	126 (33)
	Other (%)	10 (20)	140 (36)
Region[2]	Asia (%)	17 (35)	110 (29)
	Sub-Saharan Africa (%)	15 (31)	144 (38)
	Latin America (%)	7 (14)	40 (10)
	Other (%)	9 (18)	89 (23)
Sponsorship[3]	Government or academic (%)	26 (53)	187 (49)
	Foundation (%)	21 (43)	135 (35)
	Pharmaceutical or device company (%)	3 (6)	17 (4)
Analysis	National or regional scope (%)	33 (67)	262 (68)
	GDP cutoffs used (%)[4]	40 (82)	259 (67)
	Health care payer perspective (%)[5]	25 (51)	235 (61)
	Published after 2010 (%)	27 (55)	230 (60)
	GHCEA Registry Rating - mean (sd)	5.2 (1.1)	4.9 (1.1)

[1]Interventions are presented as mutually exclusive. [2]Categories are not mutually exclusive, as articles can present more than one ICER. [3]Articles could have multiple sponsors or no sponsors. [4]An intervention was deemed cost-effective if its ICER fell below a multiple of per capita GDP. [5]CEA was conducted from a healthcare payer perspective, according to the GHCEA Registry reader.
