# Supplementary material for: When cost-effective interventions are unaffordable: Integrating cost-effectiveness and budget impact in priority setting for global health programs
Source: PLoS Med. 2017 Oct 2;14(10):e1002397. doi: 10.1371/journal.pmed.1002397 (PMC5624570; doi:10.1371/journal.pmed.1002397)
Supplement: S2 Text — (PDF) [file pmed.1002397.s003.pdf]

# S2 Text: Examples of classifications in Figure 1

A Bilinski, P Neumann, J Cohen, T Thorat, K McDaniel, JA Salomon

- **Theoretically affordable:**
  - "The budgetary implications of adopting artesunate for routine use in hospital-based care are negligible." (Lubell 2011)
  - "The results of this analysis were submitted to the Armenian Ministry of Health, and led to a decision to apply for support from GAVI for rotavirus vaccine introduction." (Jit 2011)
- **Implemented:** "Cost was a major factor when the standardised programme was first implemented in 1997, with the regimen chosen being the most affordable in the context of the National Tuberculosis Programme's budget. Other options remain more expensive, but are becoming increasingly realistic with substantial reductions in drug prices." (Suárez 2002)
- **Cost barriers identified:**
  - "On the one hand, given the urgency with which the government would like to control this important public health problem, the immunization program might be able to secure the increased budget. On the other hand, the affordability of adopting this vaccine is contested at this time due to the serious economic crisis facing the country." (Aguilar 2015)
  - "We note that a fully universal plan of coverage for all 3 categories of treatment, while cost-effective, would generate a total societal cost of $13.6 billion per year (Rs. 873 billion or 87 300 crore; $2.6 billion for primary prevention, $0.8 billion for secondary prevention, and $10.2 billion for tertiary treatment), most of which might have to be borne by the government to finance national coverage, which is far larger than the current $4 billion government healthcare budget; India remains among the countries with the lowest spending as a proportion of GDP (4%), despite its growing economy." (Basu 2015)
