# Supplementary material for: When cost-effective interventions are unaffordable: Integrating cost-effectiveness and budget impact in priority setting for global health programs
Source: PLoS Med. 2017 Oct 2;14(10):e1002397. doi: 10.1371/journal.pmed.1002397 (PMC5624570; doi:10.1371/journal.pmed.1002397)
Supplement: S3 Text — (PDF) [file pmed.1002397.s004.pdf]

S3 Text: Derivation of stylized HPV vaccine example

A Bilinski, P Neumann, J Cohen, T Thorat, K McDaniel, JA Salomon

While our HPV example is simplified, it is intended to reflect the general disease dynamic:

- **Average budget:** Using the WHO Immunization Financing Indicators ("jrf_ifindictor_04.csv"), we took the mean of the amount spent by the government on vaccinations in 2015 in the AFRO region, which was approximately $9 million.
- **Costs:** We estimated costs from Quentin (2012). Based on the total economic cost of $26.41 per girl for approximately 50,000 girls, we assumed that each girl contributed $10 to overhead costs, for a total of $500,000. We multiplied this total by 1.5 to reflect the larger population in our sample, for a total of $750,000 in fixed costs. We assumed an incremental economic cost (i.e. variable cost per patient) of approximately $15/fully vaccinated girl ($10 in non-vaccine costs and a $5 vaccine cost). We assumed that 100,000 girls were vaccinated per year.
- **Benefits:** We used Tanzanian incidence of cervical cancer by age, reported in Campos (2016) (Supporting Information, Table D). We assumed that benefits were proportional to the number of vaccinated women in an age group, multipled by the cervical cancer incidence in that age group. We scaled benefits to obtain an ICER in a reasonable range (e.g. Kim (2013), Fesenfeld (2013)).
